# One New Species and Four New Records of the Genus *Amaloxestis* Gozmány (Lepidoptera: Lecithoceridae) from China: Integrative Taxonomic Evidence †

**DOI:** 10.3390/ani16091288

**Published:** 2026-04-22

**Authors:** Mian Huang, Shuhui Li, Shuai Yu

**Affiliations:** College of Agriculture and Biology, Liaocheng University, Liaocheng 252059, China; huangmian@lcu.edu.cn (M.H.); shuhuili1212@163.com (S.L.)

**Keywords:** *Amaloxestis*, Lecithocerinae, taxonomy, DNA barcode

## Abstract

The family Lecithoceridae represents one of the most diverse yet understudied groups within Lepidoptera, with numerous unresolved taxonomic problems that require urgent attention. This study reports one new species and four newly recorded species of the genus *Amaloxestis* Gozmány, 1973 from China. *Amaloxestis similinepalensis* Yu is described as new to science, while *A. astringens* Gozmány, 1973, *A. callitricha* (Meyrick, 1910), *A. chiloptila* (Meyrick, 1921) and *A. nepalensis* Gozmány, 1973 are newly recorded from China. All treated species were identified based on morphological characters and molecular phylogenetic evidence. Additionally, the females of *A. chiloptila* and *A. nepalensis* are described for the first time.

## Simple Summary

The family Lecithoceridae is one of the most species-rich families in Lepidoptera, comprising more than 1600 described species worldwide. Among its genera, *Amaloxestis* Gozmány has received remarkably limited scientific attention, with the last formal taxonomic treatment published in 1997. Furthermore, no molecular data for the genus have been available in a public database to date. In the present study, we address these critical knowledge gaps by providing comprehensive DNA barcode sequences for most known *Amaloxestis* species. We also describe one new species identified using an integrative approach that combines morphological evidence and DNA barcoding. Previously, only one *Amaloxestis* species was known from China; our research shows that six of the seven valid species in the genus are distributed in China, including the new species described in this paper. This discovery highlights the remarkably high species diversity of *Amaloxestis* in China, underscoring the country’s significance as a key hotspot for lecithocerid moth diversity.

## 1. Introduction

Lecithoceridae Le Marchand, 1947 is the sixth-largest family of the superfamily Gel-echioidea (Lepidoptera), comprising more than 1600 described species based on the author’s statistic. The family currently comprises four accepted subfamilies: Lecithocerinae, Torodorinae, Crocanthinae and Ceuthomadarinae [1,2].

*Amaloxestis* is a relatively small genus in the subfamily Lecithocerinae, established by Gozmány with *Homaloxestis callitricha* Meyrick, 1910 from India as its type species [3]; in the same paper, Gozmány transferred two additional Indian species from *Homaloxestis* to *Amaloxestis*: *H. chiloptila* Meyrick, 1921 and *H. perizeucta* Meyrick, 1910. Subsequently, Gozmány described two further new species from Nepal: *A. astringens* and *A. nepalensis* [4]. Wu later added *A. cnecosa* as a new species from China [5]. To date, six *Amoloxestis* species have been described worldwide, with only one species previously recorded from China.

*Amoloxestis* is characterized by the following features: Adult: Antenna nearly as long as forewing. Labial palpus with second palpomere thickened, third palpomere slender; male second palpomere roughly scaled or with diffused hairs dorsally. Forewing usually with a discal and a discocellular stigmata; R_1_, R_2_ free, R_3_, R_4_ and R_5_ stalked, M_2_, M_3_ stalked, CuA_1_ and CuA_2_ separated. Hindwing with Rs and M_1_ stalked, M_2_ present; M_3_ and CuA_1_ stalked, CuA_2_ free. Abdominal tergites without zones of spiniform setae. Male genitalia: Uncus lobe short. Valva broad at base; cucullus with short, strong setae above ventral margin; costal bar narrow. Juxta with the posterior lobe present. Aedeagus usually with dorsal denticles; cornuti polymorphic. Female genitalia: Apophyses posteriores longer than apophyses anteriores. Antrum cup-shaped. Ductus bursae short, sometimes with spines. Corpus bursae elliptical; signum with denticles. The genus is similar to the genus *Lecithocera* Herrich-Schäffer, 1853 in appearance and male genitalia. It can be distinguished by the combination of the forewing with M_2_ and M_3_ stalked, CuA_1_ and CuA_2_ separated and the labial palpus with long hairs on the second palpomere.

The present paper aims to describe a new species, to update the known diversity of *Amaloxestis* in China, and to provide a key to the species of the genus.

## 2. Materials and Methods

**Figure 1:**
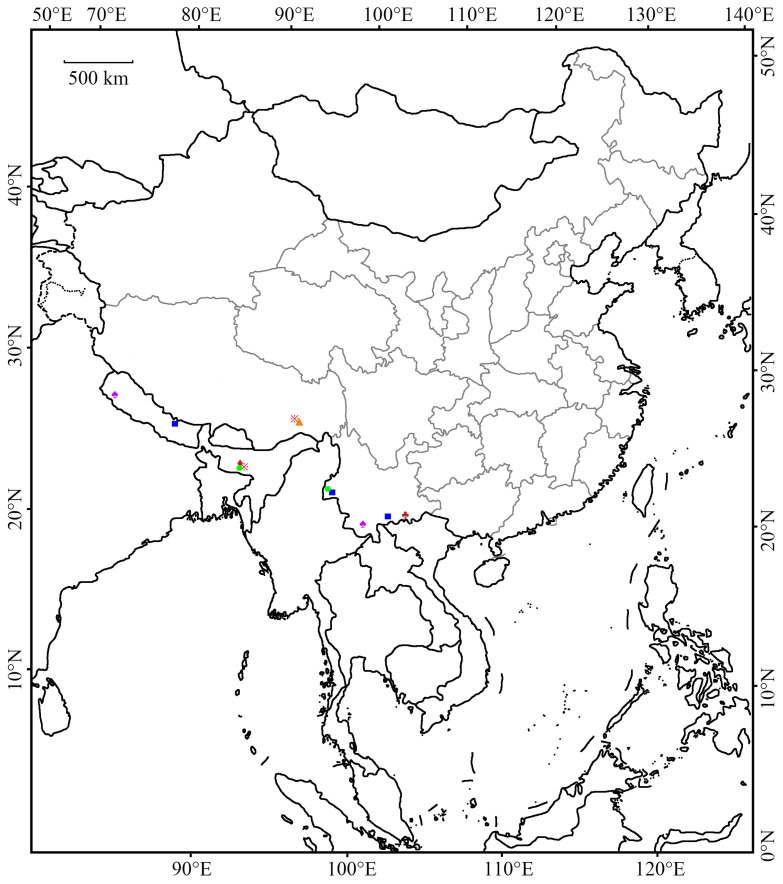
Geographic distribution of *Amaloxestis* species. Note: Symbols correspond to species as follows: ♠ *A. astringens*; ♦ *A. perizeucta*; ※ *A. chiloptila*; ■ *A. nepalensis*; ♣ *A. cnecosa*; ▲ *A. similinepalensis* Yu, sp. nov.; ● *A. callitricha*.

The examined specimens were collected using GYZ 450 W high-pressure mercury lamps (Yaming, Shanghai, China). Morphological terminology used in the descriptions follows Gozmány [6]. Wingspan measurements were taken from the tips of the left and right forewings of fully well-spread specimens. Wing venation slides and genitalia slides were prepared following the methods introduced by Li [7]. Photographs of adults were captured using an M205A stereomicroscope, and genitalia photographs were taken using a DM750 microscope with Leica Application Suite software version 4.6 (Leica, Wetzlar, Germany). All images were processed with Photoshop CC (Adobe, San Jose, CA, USA). The type series of the new species is deposited at Liaocheng University (LCU), Liaocheng, China. The distribution of *Amaloxestis* species is illustrated in Figure 1.

In this study, a total of 17 *Amaloxestis* specimens were independently collected for molecular analysis. These included three specimens of *Amaloxestis similinepalensis* Yu, sp. nov. (Vouchers: LCU243, LCU415 and YUS073), three of *A. chiloptila* (Vouchers: LCU376, LCU377 and LCU378), three of *A. astringens* (Vouchers: LCU407, LCU414 and YUS071), four of *A. nepalensis* (Vouchers: LCU404, LCU408, LCU409 and YUS074), and three of *A. callitricha* (Vouchers: LCU061, LCU405 and LCU413). Two specimens representing two *Lecithocera* species, *Lecithcera* sp. and *L. tylobathra*, were included as outgroups (Vouchers: NKU-WQY0066 and NKU-WQY0106).

Genomic DNA was extracted from the legs or partial body of dried specimens using a Genomic DNA Extraction Kit (Tiangen Biotech, Beijing, China). One mitochondrial marker (Cytochrome oxidase subunit 1 [COI]) was amplified using polymerase chain reaction (PCR). The primer used was sourced from previous studies: LCO1490/HCO2198 [8]. The sequences were manually edited using BioEdit v.7.2.5 [9] and analyzed with MEGA X (version 10.2) [10]. The gene was aligned using PhyloSuite v1.2.2 [11]. Phylogenetic reconstructions of these species were performed based on this dataset using Bayesian Inference (BI) in MrBayes 3.2 [12]. The best-fit model of sequence evolution was selected using the Akaike Information Criterion (AIC) in PartitionFinder v2 [13]. GTR + G model was selected. Four Markov chain Monte Carlo (MCMC) runs with four chains were performed for 10,000,000 generations, sampling every 1000 trees and discarding the first 25% as burn-ins.

## 3. Results

### 3.1. Molecular Analysis Results

The gene sequences generated in this study have been deposited in GenBank under the following accession numbers: PZ239923-PZ239939 (COI).

**Figure 2:**
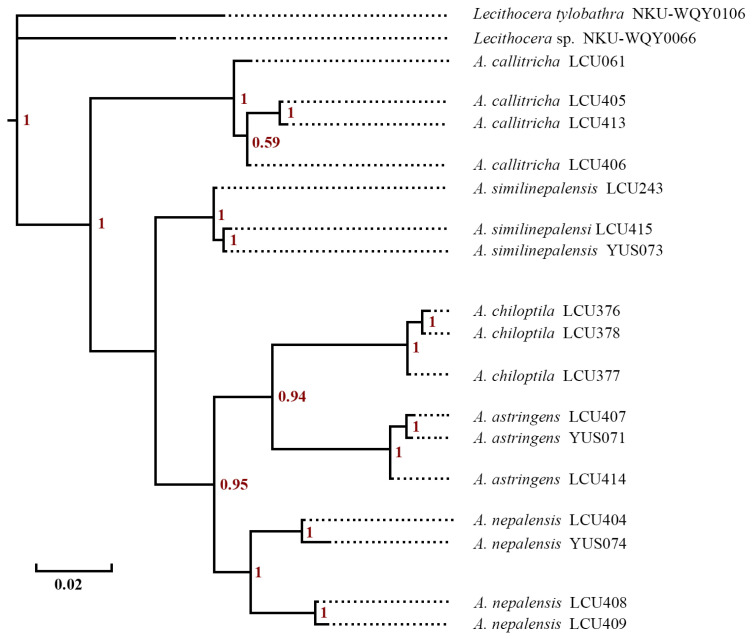
BI tree based on DNA barcodes of 17 *Amaloxestis* and 2 *Lecithocera* exemplar specimens. Numbers above branches indicate posterior probability (PP).

The Bayesian Inference (BI) tree was constructed based on 19 exemplars, and the topological results presented nearly identical results (Figure 2). The phylogenetic tree revealed the presence of six well-supported clades; each clade represents one species except the outgroup clade. The genetic distances are presented in Table 1. Intraspecific genetic distances were consistently low among species of *Amaloxestis*, ranging from 0.0020 (in *A. similinepalensis* Yu, sp. nov.) to 0.0053 (in *A. callitricha*). These values fall well below the 0.02 threshold commonly used as a cutoff for intraspecific divergence in Lepidoptera DNA barcoding studies. *Amaloxestis nepalensis* represented the only exception, with a relatively higher intraspecific distance of 0.0273, which is potentially indicative of underlying population structure or recent evolutionary divergence within this lineage. Interspecific distances were markedly higher than intraspecific values, confirming clear genetic separation among the five species. All interspecific distances exceeded 0.05, aligning with patterns of species-level differentiation observed in most insect taxa. The maximum intraspecific distance (0.0273 in *A. nepalensis*) was substantially lower than the minimum interspecific distance (0.0585 between *A. similinepalensis* Yu, sp. nov. and *A. nepalensis*), revealing a distinct barcoding gap. This gap supports the taxonomic validity of all five *Amaloxestis* species as independent evolutionary lineages, consistent with their current morphological classification.

### 3.2. Morphological Results

*Amaloxestis* Gozmány, 1971
*Amaloxestis* Gozmány, 1971, *Acta Zool. Acad. Sci. Hung.*, 17 (3–4): 251 [3]. Type species: *Homaloxestis callitricha* Meyrick, 1910.
Key to *Amaloxestis* species based on the male adult.

1. Cucullus with a process at dorsobasal corner; Costal bar with an extension dorsomedially
                                                                                                             *A. callitricha*

- Cucullus without process; Costal bar without extension            2

2 Cucullus narrowed from base to apex, its costal margin gently arched postmedially
                                                                                                             *A. chiloptila*

- Cucullus uniformly wide medially, its costal margin straight or concaved postmedially
                                                                                                              3

3 Aedeagus without dorsal denticle; cucullus concaved postmedially on costal margin
                                                                                                             *A. cnecosa*

- Aedeagus with dorsal denticles; cucullus straight postmedially on costal margin
                                                                                                              4

4 Cucullus nearly as wide as basal part of valva                            *A. astringens*

- Cucullus narrower than basal part of valva                                  5

5 Gnathos basal plate straight on posterior margin; Adult large-sized (20.0–21.0 mm)
                                                                                                             *A. perizeucta*

- Gnathos basal plate arched on posterior margin; Adult small-sized (13.0–17.0 mm)
                                                                                                              6

6 Aedeagus with more than 20 cornuti                                            *A. similinepalensis* Yu, sp. nov.

- Aedeagus with two cornuti                                                            *A. nepalensis*

#### 3.2.1. *Amaloxestis astringens* Gozmány, 1973

**Figure 3:**
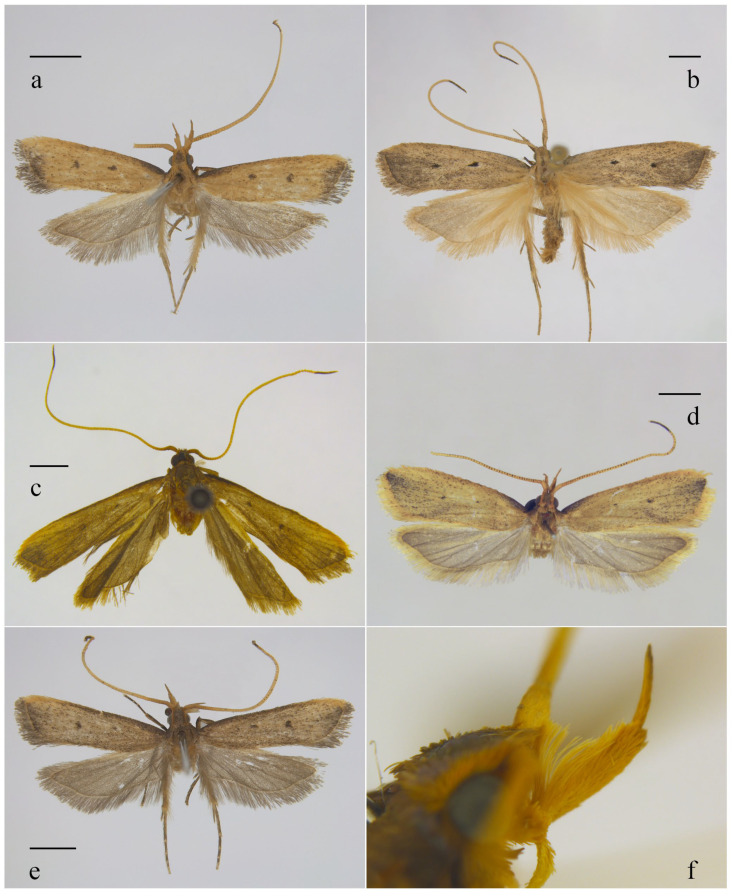
Adults of *Amaloxestis* spp. (**a**) *A. astringens*, male; (**b**) *A. callitricha*, male; (**c**) *A. chiloptila*, male; (**d**) *A. nepalensis*, male; (**e**) *A. similinepalensis* Yu, sp. nov., male, holotype, slide no. YUS073; (**f**) labial palpus of *A. similinepalensis* Yu, sp. nov. Scales = 2.0 mm.

**Figure 4:**
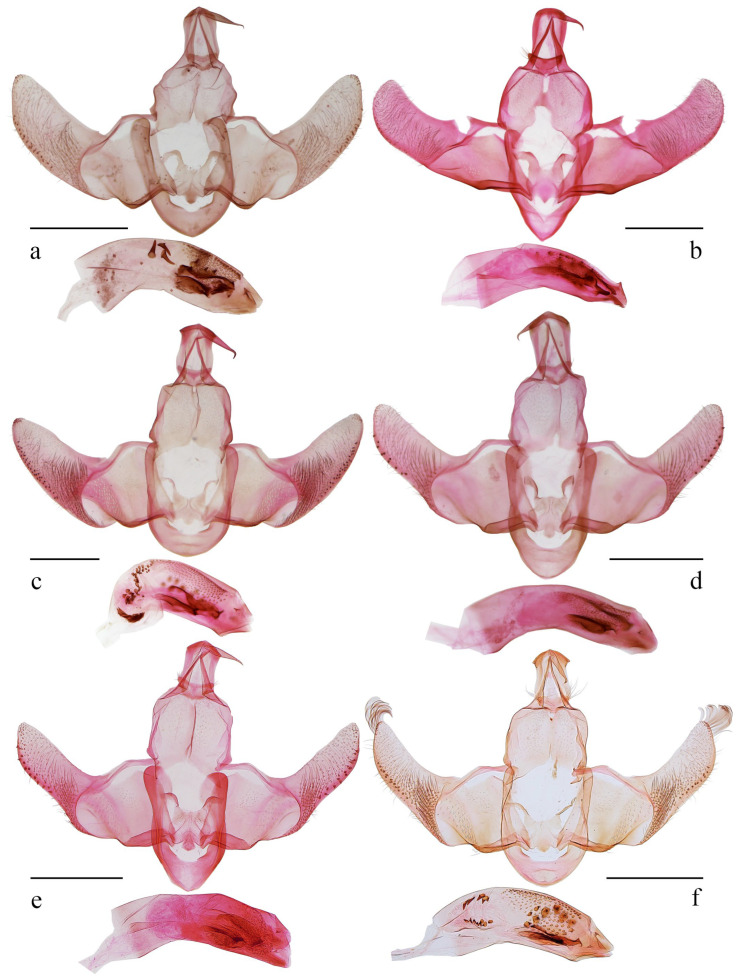
Male genitalia of *Amaloxestis* spp. (**a**) *A. astringens*, slide no. YUS071; (**b**) *A. callitricha*, slide no. LCU405; (**c**) *A. chiloptila*, slide no. LCU376; (**d**) *A. nepalensis*, slide no. LCU408; (**e**) *A. nepalensis*, slide no. YS19596; (**f**) *A. similinepalensis* Yu, sp. nov., holotype, slide no. YUS073. Scales = 0.5 mm.

*Amaloxestis astringens* Gozmány, 1973, *Khumbu Himal*., 4 (3): 417 [4]. TL: Nepal. TD: ZSM (Figure 3a and Figure 4a).

Material examined: China: 2
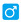

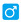
, Yunnan Prov., Xishuangbanna, Menghai, Nabanhe, 810 m, 2 Aug. 2022, leg. S. Yu & K.J. Teng, slide no. YUS071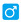, deposited in LCU; 1
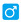

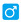
, Yunnan Prov., Xishuangbanna, Menghai, Nabanhe, 1210 m, 4 Aug. 2022, leg. S. Yu & K.J. Teng, slide no. LCU407.

Adult wingspan: 12.5–14.0 mm.

Distribution. China (Yunnan, new record), Nepal.

Note. This species was first described in Nepal based on male specimens. Herein, we report the first record of this species from China, and its female is still unstudied. The wingspan of the type series is 14.0–17.0 mm.

#### 3.2.2. *Amaloxestis callitricha* (Meyrick, 1910)

*Homaloxestis callitricha* Meyrick, 1910, *J. Bombay nat. Hist. Soc.*, 20 (2): 440 [14]. TL: India. TD: NHMUK.

*Amoloxestis callitricha* (Meyrick): Gozmány, 1971, *Acta Zool. Acad. Sci. Hung.*, 17 (3–4): 251 [3] (Figure 3b and Figure 4b).

Material examined: China: 3
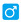

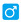
, Yunnan Prov., Yingjiang County, Tongbiguan, 2002 m, 17 Aug. 2022, leg. S. Yu, slide nos. LCU061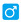, LCU405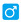, LCU406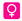, deposited in LCU.

Adult wingspan: 18.0–21.0 mm.

Distribution. China (Yunnan, new record), India.

Note. This species was first described in India. Herein, we report the first record of this species from China. The wingspan of the type series is 20.0–21.0 mm.

#### 3.2.3. *Amaloxestis chiloptila* (Meyrick, 1921)

*Homaloxestis chiloptila* Meyrick, 1921, *Exotic Microlep.*, 2 (14): 435 [15]. TL: India. TD: NHMUK.

**Figure 5:**
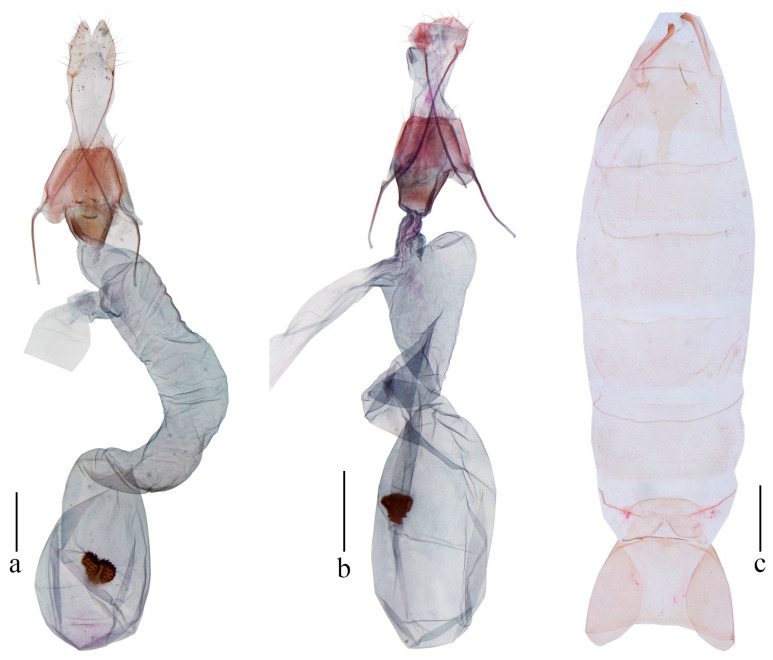
Female genitalia and abdomen of *Amaloxestis* spp. (**a**) Female genitalia of *A. chiloptila*, slide no. LCU377; (**b**) female genitalia of *A. nepalensis*, slide no. LCU409; (**c**) abdomen of *A. similinepalensis* Yu, sp. nov., holotype, slide no. YUS073. Scales = 0.5 mm.

*Amoloxestis chiloptila* (Meyrick): Gozmány, 1971, *Acta Zool. Acad. Sci. Hung.*, 17 (3–4): 252 [3] (Figure 3c, Figure 4c and Figure 5a).

Material examined: China: 3
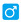

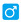
, 2
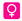

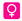
, Xizang, Motuo, Beibengxiang, 26 May 2021, leg. H.L. Han, slide nos. LCU376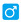, LCU377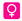, LCU378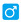, LCU379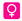, deposited in LCU.

Adult wingspan: 18.0–21.5 mm.

Female genitalia (Figure 5a): Abdominal sternite VIII gently concave medially on posterior margin. Apophyses anteriores about 3/5 the length of apophyses posteriores. Antrum sclerotized, cup-shaped. Ductus bursae broad, longer than corpus bursae; ductus seminalis broad, arising from about posterior 1/5 of ductus bursae. Corpus bursae elliptical; signum strawberry-shaped, at middle, denticulate on surface, with a longitudinal furrow along midline.

Distribution. China (Xizang, new record), India.

Note. This species was first described in India based on a male specimen. Since then, no additional specimens have been documented. Herein, we report the first record of this species from China and provide the first illustration of the female. The wingspan of the holotype is 20.0 mm.

#### 3.2.4. *Amaloxestis nepalensis* Gozmány, 1973

*Amaloxestis nepalensis* Gozmány, 1973, *Ergeb. Forsch. Nepal*, 4 (3): 418 [4]. TL: Nepal. TD: ZSM (Figure 3d, Figure 4d,e and Figure 5b).

Material examined: China: 4
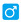

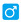
, Yunnan Prov., Lvchun County, Mt. Huanglian, 1898 m, 27 Jul. 2022, leg. S. Yu & K.J. Teng, slide no. YUS074, LCU404, deposited in LCU; 2
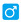

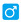
, Yunnan Prov., Mang City, Mt. Banggunjian, 1758 m, 11 Aug. 2022, leg. S. Yu, slide no. YUS072, LCU411, deposited in LCU; 2
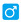

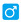
, 1
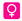

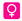
, Yunnan Prov., Yingjiang County, Tongbiguan, 1178 m; 28 Aug. 2023, leg. K.J. Teng, slide nos. LCU408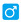, LCU409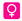, deposited in LCU; 1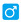, Yunnan Prov., Baoshan, Tengchong, Xiaodifang, 2116 m, 12 Aug. 2014, leg. K.J. Teng, S.R. Liu, H. Rong, slide no. YS19596, deposited in NKU; 2
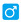

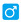
, Yunnan Prov., Baoshan, Tengchong, Xiaodifang, 2116 m, 22 Jul. 2023, leg. K.J. Teng, slide nos. LCU383, LCU410, deposited in LCU.

Adult wingspan: 14.0–17.0 mm.

Female genitalia (Figure 5b): Abdominal sternite VIII gently concave medially on posterior margin. Apophyses anteriores about 2/3 the length of apophyses posteriores. Antrum sclerotized, cup-shaped. Ductus bursae narrowed near antrum, broadened medially, as long as corpus bursae; ductus seminalis almost as wide as posterior part of ductus bursae, arising from about posterior 1/5 of ductus bursae. Corpus bursae oblong; signum strawberry-shaped, at middle, denticulate on surface.

Distribution. China (Yunnan, new record), Nepal.

Note. This species was first described from Nepal based on a male specimen. The wingspan of the holotype is 13.0 mm. Since then, no additional specimens have been documented. Herein, we report the first record of this species from China and provide the first illustration of the female. Additionally, the male genitalia of the specimen with slide number YS19596 have a slightly acute apex of the valva, which matches the characteristics of the *Amaloxestis nepalensis* holotype, whereas the specimen with slide number LCU408 has a slightly obtuse apex of the valva. We consider this to be intraspecific variation.

#### 3.2.5. *Amaloxestis similinepalensis* Yu, sp. nov.

(Figure 3e,f, Figure 4f and Figure 5c)

ZooBank registration:

Material examined: Holotype: 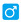, China, Xizang, Motuo County, Beibeng, 29.24° N, 95.17° E, 754 m, 14 Jun. 2023, leg. S. Yu, slide no. YUS073, deposited in LCU. Paratype: 2
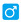

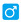
, same data as holotype, slide no. LCU243, deposited in LCU.

Diagnosis: The new species is similar to *A. nepalensis* in the wing maculation and male genitalic features, but it differs in the darker wing colour and in the aedeagus of the male genitalia with more than 20 cornuti (vs. two cornuti in *A. nepalensis*). The new species is also similar to *Amaloxestis perizeucta* (Meyrick, 1910) in male genitalia morphology, but can be distinguished by the smaller adults and the distinct blackish brown discal and discocellular stigmata of the forewing; whereas in *A. perizeucta*, the adult has a wingspan of 20.0–21.0 mm, and the forewing has indistinct discal and discocellular stigmata.

Adult (Figure 3e): Wingspan 14.5–16.0 mm. Head yellowish brown, brownish yellow along lateral sides. Antenna yellow except brownish yellow on scape and basal part of flagellum. Labial palpus yellow, third palpomere approximately 1/2 the length of second palpomere, with elongate scales along dorsal margin of second palpomere and a pencil of long hair-like scales arising dorsally from the base of second palpomere (Figure 3f). Thorax and tegula yellowish brown. Forewing brownish yellow, covered with dense dark brown scales, dark brown along costal margin from base to basal 3/4 and along termen; discal stigma dot-like; discocellular stigma larger than discal stigma, rounded; fringe grey. Hindwing grey; fringe grey with a pale-coloured basal line.

Male genitalia (Figure 4f). Uncus short, subtriangular, gently concave on posterior margin. Gnathos with basal plate arched on posterior margin, median process straight except angled near apex. Valva wide basally, sinuate ventrally; cucullus wide at base, uniformly wide medially, narrowed distally to a blunt apex, almost straight on costal margin except at base; costal bar slender, curved at basal 1/3; sacculus band-shaped. Juxta nearly trapezoidal, gently concave on posterior margin, with a semi-ovate extension at middle on anterior margin; posterolateral lobe slender and arched. Vinculum U-shaped, forming a broad saccus region anteriorly. Aedeagus stout, almost uniformly wide, with numerous disc-shaped particles distally, with two small denticles before apex; cornuti consisting of more than 10 conic spines anteriorly, and several thumbtack-shaped spines and two bars distally.

Female unknown.

Distribution: China (Xizang).

Etymology: The specific epithet is derived from the Latin *simil*- and *nepalensis*, referring to the similarity of the new species and *A. nepalensis*.

## 4. Discussion

*Macrotona* is a small genus established by Meyrick, in which four species were originally described [16]. It was later synonymized with *Lecithocera* by Meyrick, and all its species were transferred to that genus [17]. Gozmány subsequently synonymized *Macrotona* with *Amaloxestis* [6] but did not address the taxonomic placement of its constituent species. Wu followed Gozmány’s treatment [5]. Nye and Fletcher noted that *Macrotona* is a junior homonym of *Macrotona* Brunner, 1893 (Orthoptera, Acrididae), and that its type species, *M. sobria* Meyrick, 1904, is congeneric with *Carcina luticornella* Zeller, 1839—the type species of *Lecithocera*—thus rendering *Lecithocera* the valid subjective replacement name for *Macrotona* Meyrick, 1904 [18]. Nielsen et al. also treated *Macrotona* as a junior synonym of *Lecithocera* and placed the four *Macrotona* species in *Lecithocera* [19], a treatment later followed by Park and Wu [20]. In the present study, we accept the taxonomic placement considering *Macrotona* a junior synonym of *Lecithocera*.

*Amaloxestis* species exhibit a distinct distribution pattern, being restricted to the Oriental Region, with a clear concentration in Southwestern China and the adjacent Himalayan areas. This region features complex topography and diverse habitats, which may drive species differentiation and endemism. The high diversity of *Amaloxestis* in Southwestern China further underscores this region’s status as one of the world’s key biodiversity hotspots [21]. Our findings also highlight the necessity of additional faunal surveys in these understudied montane regions to better elucidate the distribution and species diversity of the genus *Amaloxestis* and the family Lecithoceridae.

In species delimitation, potential conflicts between morphological and molecular evidence have been widely documented [22]. In the present study, however, morphological characters were consistent with molecular phylogenetic results, with no conflicting signals observed. Such congruence between morphology and DNA data supports the reliability of our species identification and the taxonomic treatment of the new species.

## 5. Conclusions

Using an integrative taxonomic framework that integrates morphological examination and molecular analysis, this study greatly enriches the body of research on the poorly studied genus *Amaloxestis*. Our work substantially expands the known geographical distribution of the genus in China, generates reference DNA barcode sequences for five *Amaloxestis* species to support reliable molecular identification in future studies, and formally describes a new species of the genus.

## Figures and Tables

**Table 1:** Genetic distances among *Amaloxestis* species.

	1	2	3	4	5
*A. callitricha*	0.0053 ± 0.0023				
*A. similinepalensis*	0.0788 ± 0.0108	0.0020 ± 0.0014			
*A. chiloptila*	0.1003 ± 0.0131	0.0652 ± 0.0101	0.0030 ± 0.0017		
*A. nepalensis*	0.0849 ± 0.0112	0.0585 ± 0.0088	0.0706 ± 0.0100	0.0273 ± 0.0053	
*A. astringens*	0.1035 ± 0.0129	0.0618 ± 0.0096	0.0747 ± 0.0109	0.0692 ± 0.0098	0.0030 ± 0.0017

Mean Kimura 2-parameter (K2P) genetic distances ± standard error (SE) are shown. Diagonal values = intraspecific variation; off-diagonal values = interspecific divergence. Species order: 1 *A. callitricha*, 2 *A. similinepalensis* Yu, sp. nov., 3 *A. chiloptila*, 4 *A. nepalensis*, 5 *A. astringens*.

## Data Availability

All the sequences used in this study were accessed through the GenBank database, and the accession numbers are listed in Table S1. Morphological specimens were deposited at the Insect Collection of Nankai University (NKU), Tianjin, China (NKU), and at Liaocheng University (LCU), Liaocheng, China.

## Supplementary Materials

The following supporting information can be downloaded at https://www.mdpi.com/article/10.3390/ani16091288/s1: Table S1: List of specimen information used for this study. Reference [23] is cited in Supplementary Materials.

## Abbreviations

The following abbreviations are used in this manuscript:

LCU	Liaocheng University, Liaocheng, China
NHMUK	National History Museum, London, United Kingdom
NKU	Insect collection, College of Life Sciences, Nankai University, Tianjin, China
ZSM	Zoologische Sammlung des Bayerischen Staates, München, Germany
