# Supplementary material for: One New Species and Four New Records of the Genus Amaloxestis Gozmány (Lepidoptera: Lecithoceridae) from China: Integrative Taxonomic Evidence
Source: Animals (Basel). 2026 Apr 22;16(9):1288. doi: 10.3390/ani16091288 (PMC13162603; doi:10.3390/ani16091288)
Supplement: Supplementary file 1 [file animals-16-01288-s001.zip › Supplementary table S1.pdf]

Supplementary Table S1. Species sampled for the molecular analysis.

**Species**	**Voucher**	**GenBank accession number (COI)**	**Reference**
Amaloxestis callitricha	LCU061	PZ239923	present study
Amaloxestis callitricha	LCU405	PZ239924	present study
Amaloxestis callitricha	LCU406	PZ239926	present study
Amaloxestis callitricha	LCU413	PZ239925	present study
Amaloxestis chiloptila	LCU376	PZ239930	present study
Amaloxestis chiloptila	LCU377	PZ239932	present study
Amaloxestis chiloptila	LCU378	PZ239931	present study
Amaloxestis astringens	LCU407	PZ239933	present study
Amaloxestis astringens	LCU414	PZ239935	present study
Amaloxestis astringens	YUS071	PZ239934	present study
Amaloxestis nepalensis	LCU404	PZ239936	present study
Amaloxestis nepalensis	LCU408	PZ239938	present study
Amaloxestis nepalensis	LCU409	PZ239939	present study
Amaloxestis nepalensis	YUS074	PZ239937	present study
Amaloxestis similinepalensis	LCU243	PZ239927	present study
Amaloxestis similinepalensis	LCU415	PZ239928	present study
Amaloxestis similinepalensis	YUS073	PZ239929	present study
Lecithocera sp.	NKU-WQY0066		Wang, Q.-Y. and Li, H.-H. Phylogeny of the superfamily Gelechioidea (Lepidoptera: Obtectomera), with an exploratory application on geometric morphometrics. Zool. Scr. 49 (3), 307-328. DOI: 10.1111/zsc.12407
Lecithocera tylobathra	NKU-WQY0106		Wang, Q.-Y. and Li, H.-H. Phylogeny of the superfamily Gelechioidea (Lepidoptera: Obtectomera), with an exploratory application on geometric morphometrics. Zool. Scr. 49 (3), 307-328. DOI: 10.1111/zsc.12407
